# Crystal structure of 1-meth­oxy-5-methyl-*N*-phenyl-1,2,3-triazole-4-carboxamide

**DOI:** 10.1107/S2056989015017776

**Published:** 2015-09-26

**Authors:** Inna S. Khazhieva, Tatiana V. Glukhareva, Pavel A. Slepukhin, Yury Yu. Morzherin

**Affiliations:** a Ural Federal University, Mira 19 Ekaterinburg 620002, Russian Federation; b I. Postovsky Institute of Organic Synthesis, Kovalevskoy 22 Ekaterinburg 620090, Russian Federation

**Keywords:** crystal structure, 1,2,3-triazole, rearrangements, hydrogen bonding

## Abstract

The title compound, *C*_11_H_12_N_4_O_2_,was prepared *via* the transformation of sodium 4-acetyl-1-phenyl-1*H*-[1.2.3]triazolate under the action of meth­oxy­amine hydro­chloride. The dihedral angle between the triazole and phenyl rings is 25.12 (16)° and the C atom of the meth­oxy group deviates from the triazole plane by 0.894 (4)Å. The conformation of the CONH*R*-group is consolodated by an intra­molecular N—H⋯N hydrogen bond to an N-atom of the triazole ring, which closes an *S*(5) ring. In the crystal, weak N—H⋯N hydrogen bonds link the mol­ecules into *C*(6) [010] chains.

## Related literature   

For biological activities of 1.2.3-triazoles, see: Sathish Kumar & Kavitha (2013 ▸); Khazhieva *et al.* (2015*a* ▸). For the synthesis, see: Khazhieva *et al.* (2015*b* ▸).
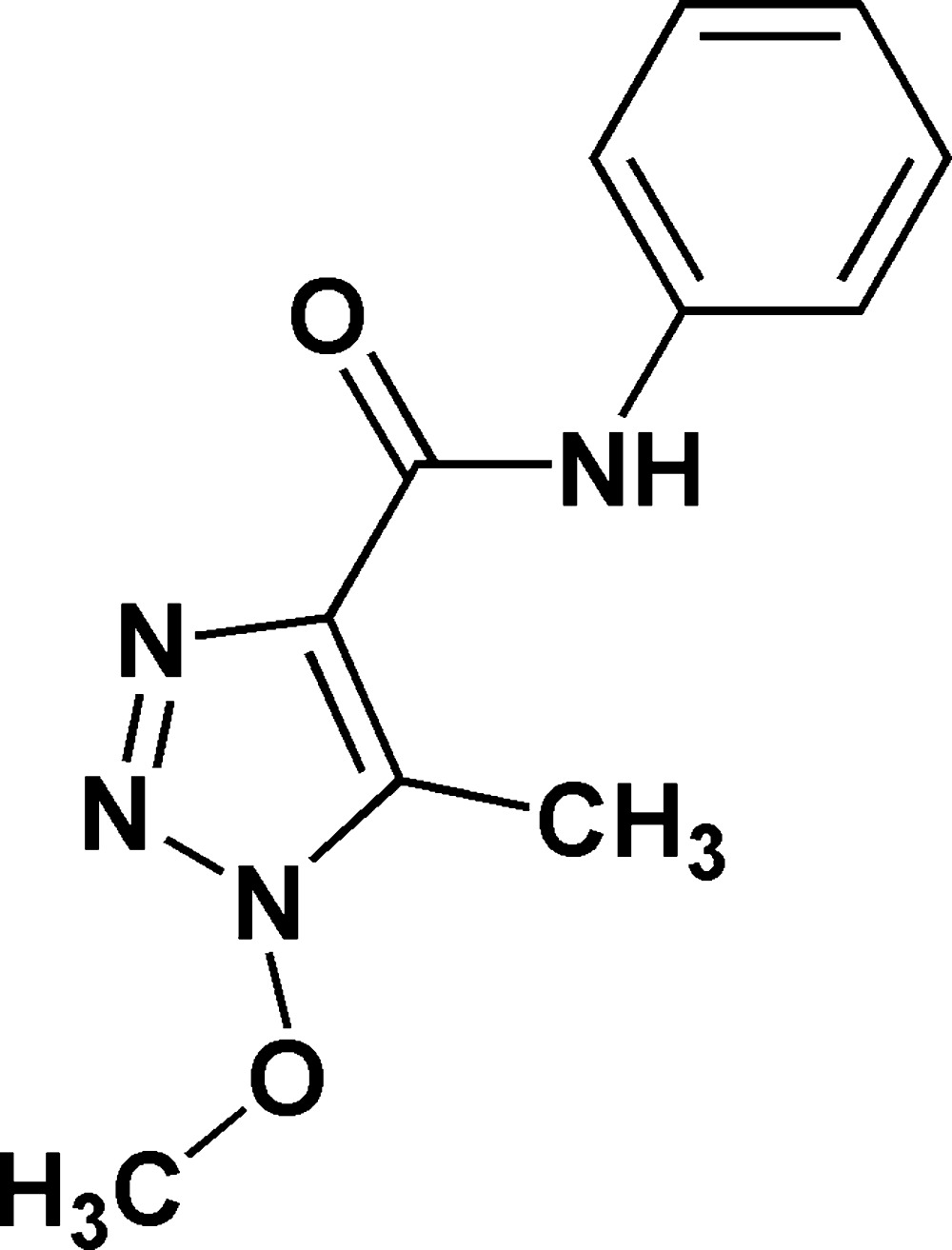


## Experimental   

### Crystal data  

C_11_H_12_N_4_O_2_
*M* *_r_* = 232.25
Monoclinic, 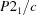

*a* = 11.4637 (8) Å
*b* = 6.4345 (13) Å
*c* = 15.822 (3) Å
β = 100.367 (12)°
*V* = 1148.0 (3) Å^3^
*Z* = 4
Mo *K*α radiation
μ = 0.10 mm^−1^
*T* = 295 K
0.21 × 0.16 × 0.09 mm

### Data collection  

Agilent Xcalibur S CCD diffractometer
7259 measured reflections
2302 independent reflections
1077 reflections with *I* > 2σ(*I*)
*R*_int_ = 0.040

### Refinement  

*R*[*F* ^2^ > 2σ(*F* ^2^)] = 0.055
*wR*(*F* ^2^) = 0.147
*S* = 1.00
2302 reflections
160 parameters
H atoms treated by a mixture of independent and constrained refinement
Δρ_max_ = 0.43 e Å^−3^
Δρ_min_ = −0.22 e Å^−3^

### 

Data collection: *CrysAlis PRO* (Agilent, 2006 ▸); cell refinement: *CrysAlis PRO*; data reduction: *CrysAlis PRO*; program(s) used to solve structure: *SHELXS97* (Sheldrick, 2008 ▸); program(s) used to refine structure: *SHELXS97* (Sheldrick, 2008 ▸); molecular graphics: *publCIF* (Westrip, 2010 ▸); software used to prepare material for publication: *publCIF* (Westrip, 2010 ▸).

## Supplementary Material

Crystal structure: contains datablock(s) I, exp_221. DOI: 10.1107/S2056989015017776/hb7511sup1.cif

Structure factors: contains datablock(s) I. DOI: 10.1107/S2056989015017776/hb7511Isup2.hkl

Supporting information file. DOI: 10.1107/S2056989015017776/hb7511Isup3.cml

**Fig. 1.**
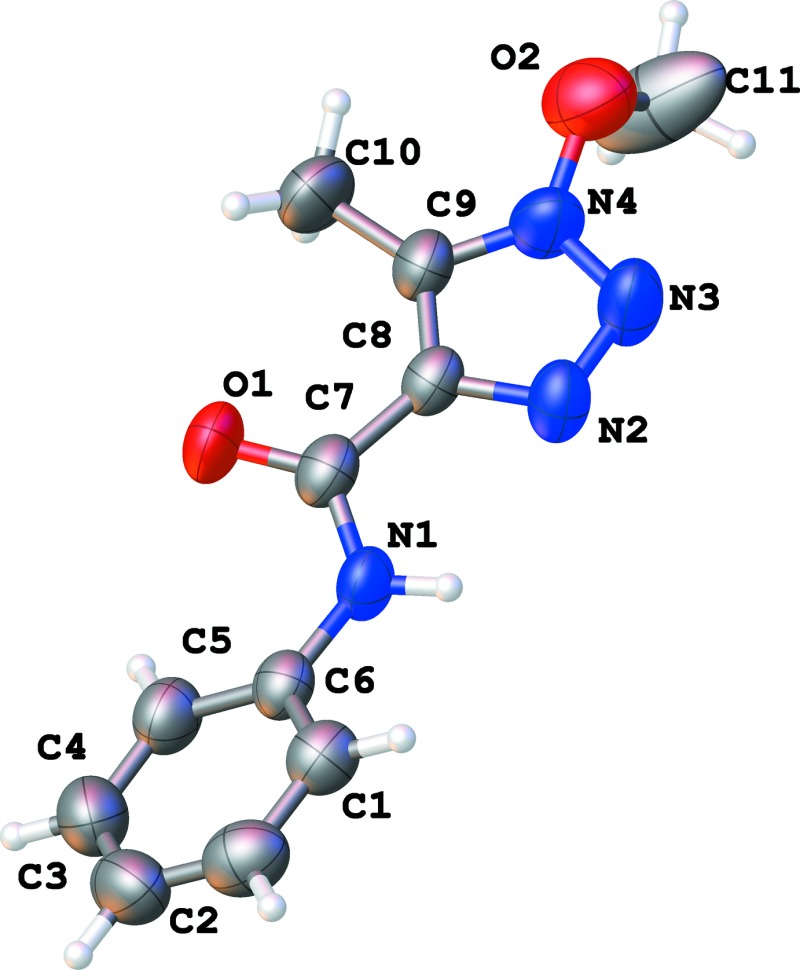
The molecular structure of (I), with 50% probability displacement ellipsoids for non-H atoms.

. DOI: 10.1107/S2056989015017776/hb7511fig1.tif
The mol­ecular structure of (I), with 50% probability displacement ellipsoids for non-H atoms.

CCDC reference: 1426448

Additional supporting information:  crystallographic information; 3D view; checkCIF report

## Figures and Tables

**Table 1:** Hydrogen-bond geometry (, )

*D*H*A*	*D*H	H*A*	*D* *A*	*D*H*A*
N1H1N2	0.86(2)	2.33(3)	2.780(4)	113(2)
N1H1N3^i^	0.86(2)	2.41(2)	3.184(3)	150(2)

Symmetry code: (i) 
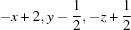
.

## supplementary crystallographic information

### S1. Synthesis and crystallization

The titled compound was prepared as previously reported (Khazhieva *et al.*, 2015*b*). Crystals were obtained by slow evaporation of a solution in ethanol.

### Crystal data

**Table:**

C_11_H_12_N_4_O_2_	*D*_x_ = 1.344 Mg m^−^^3^
*M**_r_* = 232.25	Melting point: 310 K
Monoclinic, *P*2_1_/*c*	Mo *K*α radiation, λ = 0.71073 Å
*a* = 11.4637 (8) Å	Cell parameters from 1077 reflections
*b* = 6.4345 (13) Å	θ = 2.9–26.4°
*c* = 15.822 (3) Å	µ = 0.10 mm^−^^1^
β = 100.367 (12)°	*T* = 295 K
*V* = 1148.0 (3) Å^3^	Prism, colorless
*Z* = 4	0.21 × 0.16 × 0.09 mm
*F*(000) = 488	

### Data collection

**Table:**

Agilent Xcalibur S CCD diffractometer	1077 reflections with *I* > 2σ(*I*)
Radiation source: fine-focus sealed tube	*R*_int_ = 0.040
Graphite monochromator	θ_max_ = 26.4°, θ_min_ = 2.9°
ω scans	*h* = −7→14
7259 measured reflections	*k* = −5→8
2302 independent reflections	*l* = −19→19

### Refinement

**Table:**

Refinement on *F*^2^	Primary atom site location: structure-invariant direct methods
Least-squares matrix: full	Secondary atom site location: difference Fourier map
*R*[*F*^2^ > 2σ(*F*^2^)] = 0.055	Hydrogen site location: inferred from neighbouring sites
*wR*(*F*^2^) = 0.147	H atoms treated by a mixture of independent and constrained refinement
*S* = 1.00	*w* = 1/[σ^2^(*F*_o_^2^) + (0.0682*P*)^2^] where *P* = (*F*_o_^2^ + 2*F*_c_^2^)/3
2302 reflections	(Δ/σ)_max_ < 0.001
160 parameters	Δρ_max_ = 0.43 e Å^−^^3^
0 restraints	Δρ_min_ = −0.22 e Å^−^^3^

### Special details

**Table:**

Geometry. All e.s.d.'s (except the e.s.d. in the dihedral angle between two l.s. planes) are estimated using the full covariance matrix. The cell e.s.d.'s are taken into account individually in the estimation of e.s.d.'s in distances, angles and torsion angles; correlations between e.s.d.'s in cell parameters are only used when they are defined by crystal symmetry. An approximate (isotropic) treatment of cell e.s.d.'s is used for estimating e.s.d.'s involving l.s. planes.
Refinement. Refinement of *F*^2^ against ALL reflections. The weighted *R*-factor *wR* and goodness of fit *S* are based on *F*^2^, conventional *R*-factors *R* are based on *F*, with *F* set to zero for negative *F*^2^. The threshold expression of *F*^2^ > σ(*F*^2^) is used only for calculating *R*-factors(gt) *etc*. and is not relevant to the choice of reflections for refinement. *R*-factors based on *F*^2^ are statistically about twice as large as those based on *F*, and *R*-factors based on ALL data will be even larger.

### Fractional atomic coordinates and isotropic or equivalent isotropic displacement parameters (Å^2^)

**Table:**

	*x*	*y*	*z*	*U*_iso_*/*U*_eq_	
O1	0.61315 (16)	0.0563 (3)	0.15704 (14)	0.0777 (7)	
C8	0.7887 (2)	0.1712 (4)	0.24262 (18)	0.0475 (7)	
C6	0.7399 (2)	−0.2566 (4)	0.08075 (18)	0.0496 (7)	
C7	0.7204 (2)	0.0365 (4)	0.17728 (19)	0.0533 (7)	
N2	0.90562 (18)	0.1400 (4)	0.27423 (17)	0.0605 (7)	
N4	0.8463 (2)	0.3915 (4)	0.33811 (19)	0.0708 (8)	
C9	0.7489 (2)	0.3375 (4)	0.28291 (19)	0.0553 (8)	
N1	0.7844 (2)	−0.1083 (4)	0.14353 (16)	0.0530 (6)	
N3	0.9416 (2)	0.2771 (4)	0.3343 (2)	0.0759 (8)	
O2	0.8515 (2)	0.5302 (4)	0.40535 (18)	0.0956 (8)	
C1	0.7975 (2)	−0.4450 (5)	0.0824 (2)	0.0589 (8)	
H1A	0.8634	−0.4721	0.1246	0.071*	
C5	0.6434 (3)	−0.2172 (5)	0.0169 (2)	0.0641 (8)	
H5A	0.6049	−0.0896	0.0148	0.077*	
C3	0.6605 (3)	−0.5520 (6)	−0.0411 (2)	0.0809 (10)	
H3A	0.6331	−0.6524	−0.0821	0.097*	
C2	0.7571 (3)	−0.5924 (5)	0.0214 (2)	0.0739 (9)	
H2A	0.7955	−0.7200	0.0225	0.089*	
C4	0.6048 (3)	−0.3651 (6)	−0.0430 (2)	0.0769 (10)	
H4A	0.5395	−0.3381	−0.0857	0.092*	
C11	0.9070 (4)	0.7045 (6)	0.3901 (3)	0.137 (2)	
H11A	0.8970	0.8073	0.4321	0.205*	
H11B	0.8741	0.7551	0.3337	0.205*	
H11C	0.9900	0.6765	0.3933	0.205*	
C10	0.6343 (3)	0.4512 (5)	0.2740 (2)	0.0818 (10)	
H10A	0.6298	0.5214	0.3268	0.123*	
H10B	0.5699	0.3543	0.2609	0.123*	
H10C	0.6292	0.5511	0.2284	0.123*	
H1	0.858 (2)	−0.109 (4)	0.1666 (17)	0.048 (8)*	

### Atomic displacement parameters (Å^2^)

**Table:**

	*U*^11^	*U*^22^	*U*^33^	*U*^12^	*U*^13^	*U*^23^
O1	0.0365 (12)	0.1021 (17)	0.0930 (17)	0.0057 (10)	0.0076 (11)	−0.0186 (13)
C8	0.0393 (15)	0.0458 (16)	0.0589 (18)	0.0007 (12)	0.0134 (13)	0.0056 (14)
C6	0.0414 (15)	0.0538 (18)	0.0549 (19)	−0.0035 (14)	0.0117 (14)	0.0045 (16)
C7	0.0418 (16)	0.0582 (18)	0.062 (2)	−0.0016 (14)	0.0146 (15)	0.0066 (16)
N2	0.0444 (14)	0.0516 (15)	0.0836 (19)	−0.0032 (11)	0.0062 (13)	−0.0019 (14)
N4	0.0607 (17)	0.0658 (17)	0.089 (2)	−0.0011 (13)	0.0206 (15)	−0.0268 (16)
C9	0.0400 (16)	0.065 (2)	0.0624 (19)	−0.0028 (14)	0.0117 (15)	−0.0013 (16)
N1	0.0354 (13)	0.0558 (15)	0.0661 (17)	0.0045 (11)	0.0041 (12)	−0.0011 (13)
N3	0.0465 (15)	0.0710 (17)	0.107 (2)	0.0004 (13)	0.0059 (14)	−0.0188 (17)
O2	0.0967 (18)	0.0961 (18)	0.102 (2)	−0.0125 (14)	0.0395 (15)	−0.0152 (16)
C1	0.0573 (17)	0.0563 (19)	0.064 (2)	0.0021 (15)	0.0140 (15)	0.0062 (17)
C5	0.0512 (18)	0.073 (2)	0.067 (2)	0.0069 (15)	0.0089 (16)	0.0035 (19)
C3	0.077 (2)	0.093 (3)	0.075 (3)	−0.018 (2)	0.018 (2)	−0.024 (2)
C2	0.081 (2)	0.061 (2)	0.085 (3)	−0.0020 (18)	0.030 (2)	−0.004 (2)
C4	0.061 (2)	0.098 (3)	0.070 (2)	−0.005 (2)	0.0055 (17)	−0.010 (2)
C11	0.171 (4)	0.055 (2)	0.221 (5)	−0.019 (2)	0.133 (4)	−0.005 (3)
C10	0.0566 (19)	0.098 (2)	0.092 (3)	0.0195 (17)	0.0172 (17)	−0.016 (2)

### Geometric parameters (Å, º)

**Table:**

O1—C7	1.220 (3)	C1—C2	1.372 (4)
C8—N2	1.359 (3)	C1—H1A	0.9300
C8—C9	1.365 (3)	C5—C4	1.359 (4)
C8—C7	1.463 (4)	C5—H5A	0.9300
C6—C1	1.379 (4)	C3—C4	1.360 (5)
C6—C5	1.381 (4)	C3—C2	1.370 (5)
C6—N1	1.405 (3)	C3—H3A	0.9300
C7—N1	1.354 (3)	C2—H2A	0.9300
N2—N3	1.308 (3)	C4—H4A	0.9300
N4—N3	1.327 (3)	C11—H11A	0.9600
N4—C9	1.334 (3)	C11—H11B	0.9600
N4—O2	1.382 (3)	C11—H11C	0.9600
C9—C10	1.488 (4)	C10—H10A	0.9600
N1—H1	0.85 (3)	C10—H10B	0.9600
O2—C11	1.333 (4)	C10—H10C	0.9600
			
N2—C8—C9	109.5 (2)	C4—C5—C6	120.0 (3)
N2—C8—C7	122.6 (2)	C4—C5—H5A	120.0
C9—C8—C7	127.8 (2)	C6—C5—H5A	120.0
C1—C6—C5	119.6 (3)	C4—C3—C2	120.0 (3)
C1—C6—N1	118.1 (3)	C4—C3—H3A	120.0
C5—C6—N1	122.3 (3)	C2—C3—H3A	120.0
O1—C7—N1	124.1 (3)	C3—C2—C1	120.2 (3)
O1—C7—C8	120.6 (2)	C3—C2—H2A	119.9
N1—C7—C8	115.3 (2)	C1—C2—H2A	119.9
N3—N2—C8	109.2 (2)	C5—C4—C3	120.7 (3)
N3—N4—C9	115.2 (2)	C5—C4—H4A	119.6
N3—N4—O2	118.1 (3)	C3—C4—H4A	119.6
C9—N4—O2	125.9 (2)	O2—C11—H11A	109.5
N4—C9—C8	101.5 (2)	O2—C11—H11B	109.5
N4—C9—C10	123.7 (3)	H11A—C11—H11B	109.5
C8—C9—C10	134.8 (3)	O2—C11—H11C	109.5
C7—N1—C6	126.3 (3)	H11A—C11—H11C	109.5
C7—N1—H1	113.4 (17)	H11B—C11—H11C	109.5
C6—N1—H1	120.2 (17)	C9—C10—H10A	109.5
N2—N3—N4	104.6 (2)	C9—C10—H10B	109.5
C11—O2—N4	111.1 (3)	H10A—C10—H10B	109.5
C2—C1—C6	119.6 (3)	C9—C10—H10C	109.5
C2—C1—H1A	120.2	H10A—C10—H10C	109.5
C6—C1—H1A	120.2	H10B—C10—H10C	109.5

### Hydrogen-bond geometry (Å, º)

**Table:**

*D*—H···*A*	*D*—H	H···*A*	*D*···*A*	*D*—H···*A*
N1—H1···N2	0.86 (2)	2.33 (3)	2.780 (4)	113 (2)
N1—H1···N3^i^	0.86 (2)	2.41 (2)	3.184 (3)	150 (2)

Symmetry code: (i) −*x*+2, *y*−1/2, −*z*+1/2.
