# Supplementary figures and images for: Crystal structure of 1-meth­oxy-5-methyl-N-phenyl-1,2,3-triazole-4-carboxamide
Source: Acta Crystallogr E Crystallogr Commun. 2015 Sep 26;71(Pt 10):o798. doi: 10.1107/S2056989015017776 (PMC4647442; doi:10.1107/S2056989015017776)

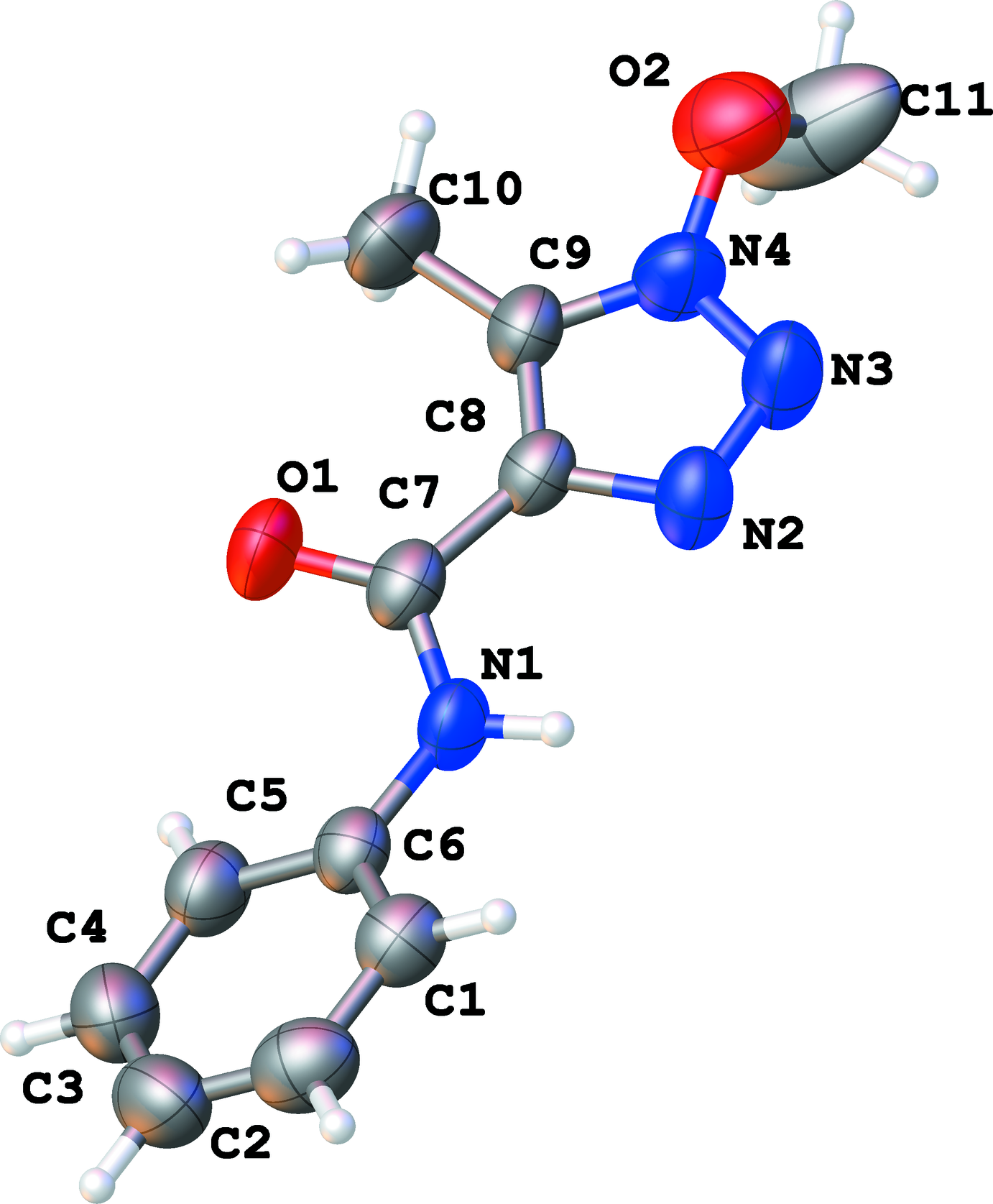

Supplement: Supplementary file 4 [file e-71-0o798-fig1.tif]
